# Profile: André Tomlin

**DOI:** 10.1192/bjb.2017.23

**Published:** 2018-04

**Authors:** Julia Bland

**Julia Bland meets André of the Elves**, arch communicator and pretension buster, man with a mission: to get healthcare workers better informed so that they can provide higher quality care to patients


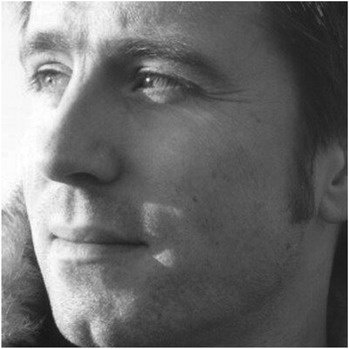


The Mental Elf is a charming woodland creature, smiling, intelligent, sensitive, balanced, with no beard, pointy ears, or a cap with a bell on the top.

In fact, he is André Tomlin, 45, information scientist, health blogger, educator, evidence-based practice guru, businessman with a genuine vision of improving the quality of healthcare, and father of three children under 8 years of age. He has over 50 000 followers of his Twitter account, and claims that the twitter community's outrage at Asda for selling mental patient costumes complete with axe and blood for Halloween, led to the removal of the offending items. He sees himself as embedded into an online community of people trying to make sense of their experience (he also has personal experience of anxiety and depression).

If you haven't heard of the National Elf Service (www.nationalelfservice.net), launched in May 2015, you may need an explanation.

This is an online subscription service that provides digests of the latest research in mental health, tailored to your interests, plus a host of interactive opportunities, like webchats, videos, and continuing professional development certificates, all ‘gamified’ with levels to achieve and gnomic cartoons dancing about. Actually, much less irritating than you might have expected.

Tomlin explained to me the genesis of the National Elf Service. He and his business partner Douglas Badenoch, worked for years at the digital end of evidence-based medicine, believing in the importance of the translation of research into practice, but were frustrated by the unappealing lumpiness of the website delivery vehicles.

They wanted to develop a user-friendly, highly accessible, but accurate and reliable online resource for the time-constrained front-line mental health worker.

Have they done this? And what else do they do? And how does it work as a business versus a piece of philanthropy?

I went to meet André Tomlin with an open mind, but holding a comment from a colleague with a key question: ‘He seems to be dominating the social media side of mental health. Why has he become so successful with this and what are his credentials in dictating the agenda?’

The unique selling point of the Mental Elf is the combination of the serious with the playful. The antithesis of pompous. I was convinced of the underlying seriousness, even idealism, of the intentions: ‘Accessibility, Usability, Reliability ….. No bias, no misinformation, no spin’, with a view to improving standards of mental health provision with a personalised feed of information to the smartphones of mental health professionals, (especially nurses) and of course, some of his subscribers are patients. His anti-silo, deliberate, democratising intention is to open up the debate around mental health. He thinks that not only should patients and their families be listened to, but also non-health professionals; for example, schools, in relation to child mental health. Social media in the mental health field has a supportive and welcoming rather than aggressive tone, he claims. For example, when the Daily Mirror outed the Everton footballer Aaron Lennon as having been sectioned, there were over a hundred tweets supporting him and criticising the Daily Mirror.

The benign aspect of the Mental Elf is also illustrated by their work with charities like Rethink, Mind, and the Mental Health Foundation. He sees the work as partly public service, running tweet chats offering peer support on topics like suicide and severe mental illness with ‘twitter buddies’, usually clinical psychologists, who can take distressed individuals aside for a private ‘direct message conversation’, and can suggest appropriate action, for example, contacting the crisis team or going to an Accident & Emergency Department.

Of course, this is also a business, and he freely admits that the line between business and altruism is a ‘blurred boundary’. He and Badenoch, another ‘information scientist’, run Minervation (an evidence-based healthcare consultancy) plus the Elves empire. They conveniently believe in ‘coproduction’. As one expert invited to write for the Mental Elf put it bluntly, ‘We don't get paid. We do the work, and he gets the money.’ Tomlin is frank: ‘Sometimes I help people, sometimes I push people towards my product, and sometimes I just have to do something to make money.’

Minervation, which ‘spun out of the university’ (i.e. the Oxford Centre for Evidence-Based Mental Health), also provides consultancy to big health players: the National Institute for Health and Care Excellence, Cochrane, and Bupa amongst them. They also specialise in building accessible websites for charities and other public sector organisations.

One of his favourite projects was for the General Medical Council. Five years ago, he developed a programme to help general practitioners provide better care for people with learning difficulties. They included a ‘forum theatre’ approach, where a play scenario is set up: a woman with Down's syndrome and her sister present at Accident & Emergency. She has stopped eating and drinking. As the play evolves, the audience can challenge and participate, ‘why is the doctor talking to her sister and not to her?’. This can then be digitally broadcast and helps promote Mencap's priority to improve communication by professionals with people with intellectual disabilities.

The Elves are accessed via subscription, and it's the institutional subscriptions that are most lucrative (over £1000 for a year for group users v. £60 per year for individuals). He has signed up universities including Oxford and Manchester, and Mental Health Trusts such as University College London, hoping to include Kings and in particular, the Masters course at the Institute of Psychiatry, Psychology and Neuroscience soon. Institutions are provided with regular anonymised reports on how their staff are using the Elf system, such as the most popular blogs, the number of reflective practice entries and the research impact.

Subscribers get access to digests of new research with comments on clinical relevance by expert reviewers, connection to other experts via online discussion and journal clubs, and a personalised ‘gamified’ continuing professional development record.

Minervation also run a digital conference service called Beyond the Room, which prepares for, attends, live streams, blogs, and podcasts from your conference for a fee. They see the slides in advance, speak to the speakers, and then do live tweeting and blogging from the conference floor, putting up podcasts afterwards. All this enables a small number physical conference to reach a far larger potential audience.

They used to offer critical appraisal workshops, but have had greater uptake for workshops teaching psychiatrists and others to use twitter and blogs. He believes all this digital output needs to be researched itself with ‘alt metrics’. He fully accepts that numbers of hits don't reflect quality: 3 years ago, the most popular blog was about dolphin semen, and he wants to improve the capacity of digital sites in ‘meaningful analytics’, which genuinely measure the effect of published articles.

Another Tomlin initiative is a public discussion with experts, every three months, linking Mental Elf, Lancet Psychiatry and University College London psychiatry. They have covered subjects like dementia, digital mental health, preventable harm and women's mental health.

They have developed the Social Care Elf, Learning Difficulties Elf, Dental Elf, Lifestyle Elf and the Diabetes Elf, and are hoping to expand into Elder Elf, Public Elf, Economist Elf, Sexual Elf and Stroke Elf.

Tomlin's background is interesting. His Dutch maternal grandfather, who just escaped being shot by Nazis twice during the occupation of the Netherlands, took young André round the red light district of Amsterdam as a boy. His parents were active members of the Campaign for Nuclear Disarmament and he ‘hung around Greenham Common’, the extensive antinuclear missile protest site, as a child.

His adult politics remain broadly left of centre but he is no dogmatist.

After philosophy and English at university he became assistant librarian at the Institute of Health Sciences in Oxford in 1993. After a Masters in Information Science at the University of West London, he returned to Oxford to help psychiatrist Professor John Geddes establish the Centre for Evidence-Based Mental Health.

Although remaining an ardent supporter of evidence-based medicine, he describes his growing impatience with the inaccessibility of the main health information websites. He and Badenoch could see how newer technology can deliver much faster and more personalised information. He also sees the speed of blogging reactions as contributing to dispelling misinformation.

The example he gives is after a paper with negative findings, determining whether exercise was useful in the treatment of depression, was taken up by the newspaper headlines as ‘Exercise found to be no help in depression’. Tomlin immediately wrote a blog publishing the actual evidence, correcting the interpretation, pointing out that exercise may still be preventative in depression, alerted the Twittersphere, and the BBC. Later that day the newspaper headlines were changed.

The power of social media operating in the interest of accuracy (for once).

Another mission is in myth busting, with evidence, of potentially harmful treatments; for example, a recent review piece in Mental Elf by Edel McGlanaghy on the subject of the potentially harmful effects of psychological treatment, mainly online cognitive–behavioural therapy for anxiety.^1^

We discussed the merits and demerits of ‘trans diagnostic’ approaches to mental healthcare, recently written about in relation to Child and Adolescent mental health by Miranda Wolpert and Peter Fonagy.^2^

They have looked at the limitations of diagnosis and evidence-based research, proposing focusing on the person rather than the diagnosis. They point to the long waits for Child and Adolescent Mental Health Services (CAMHS), and the unsatisfactory outcomes for a significant minority of patients. Wolpert recommends a practical, evidence-based coproduction approach, developing apps for young people to prepare for seeing psychiatrists.

In January 2017, the Mental Elf published a systematic review of digital interventions for young people with mental health problems. They concluded that evidence is uncertain for digital interventions and should extend rather than replace offline services, with more interdisciplinary research needed, designed with user input and involving computer scientists, and engineers as well as psychologists and psychiatrists.

This migration on Tomlin's part from traditional psychiatric research towards the ‘groovy social science side’ is never going overboard, he reassures me. ‘I'll stop in the safe middle ground’. He feels his education about psychiatry has been extended beyond ICD-10^3^ via twitter contact with patients and nurses, reaching out into the wider waters of social care and psychology. But writing from the user perspective can become flaky, he admits, and although his personal philosophy is transdiagnostic, he fully supports psychiatrists who must treat patients on particular pathways determined by diagnosis.

He is also increasingly sceptical about the evidence-based medicine world, which he sees as ‘quite insular’, and even research more broadly, claiming that about half published research is methodologically unsound, and another half never reaches publication because of the bias against publishing negative findings. In fact, he goes even further in pointing to the gap between evidence-based medicine and ordinary practitioners, and states ‘we would have most impact if we just implemented what we know now.’

So, is he a breath of fresh modernising air that we psychiatrists should breathe in deeply, and are sorely in need of with our rushed lives, or is the Mental Elf a bit gimmicky or presumptuous in setting the tone and agenda for public debate?

You will need to visit the Mental Elf site (free month's trial) and decide for yourself.

## About the author

**Julia Bland** is an honorary consultant psychiatrist for South London and Maudsley NHS Foundation Trust and a consultant medical psychotherapist at DocHealth, British Medical Association, UK; email: julia.bland@hotmail.co.uk
